# Supplementary material for: The folding and unfolding behavior of ribonuclease H on the ribosome
Source: J Biol Chem. 2020 Jun 11;295(33):11410–7. doi: 10.1074/jbc.RA120.013909 (PMC7450101; doi:10.1074/jbc.RA120.013909)
Supplement: Supporting Information [file supp_295_33_11410__index.html]

The folding and unfolding behavior of ribonuclease H on the ribosome — Folding and unfolding behavior of RNase H on the ribosome — The folding and unfolding behavior of ribonuclease H on the ribosome — EDITORS' PICK: Folding and unfolding behavior of RNase H on the ribosome — Supporting Information 

# The folding and unfolding behavior of ribonuclease H on the ribosome

## Supporting Information

- Supporting Information (to be published online) - Supplementary Figures 1 and 2 containing gels from pulse proteolysis.
