# Supplementary material for: The folding and unfolding behavior of ribonuclease H on the ribosome
Source: J Biol Chem. 2020 Jun 11;295(33):11410–7. doi: 10.1074/jbc.RA120.013909 (PMC7450101; doi:10.1074/jbc.RA120.013909)
Supplement: Supporting Information [file supp_RA120.013909_160199_2_supp_542456_qbywxs.pdf]

Supporting information

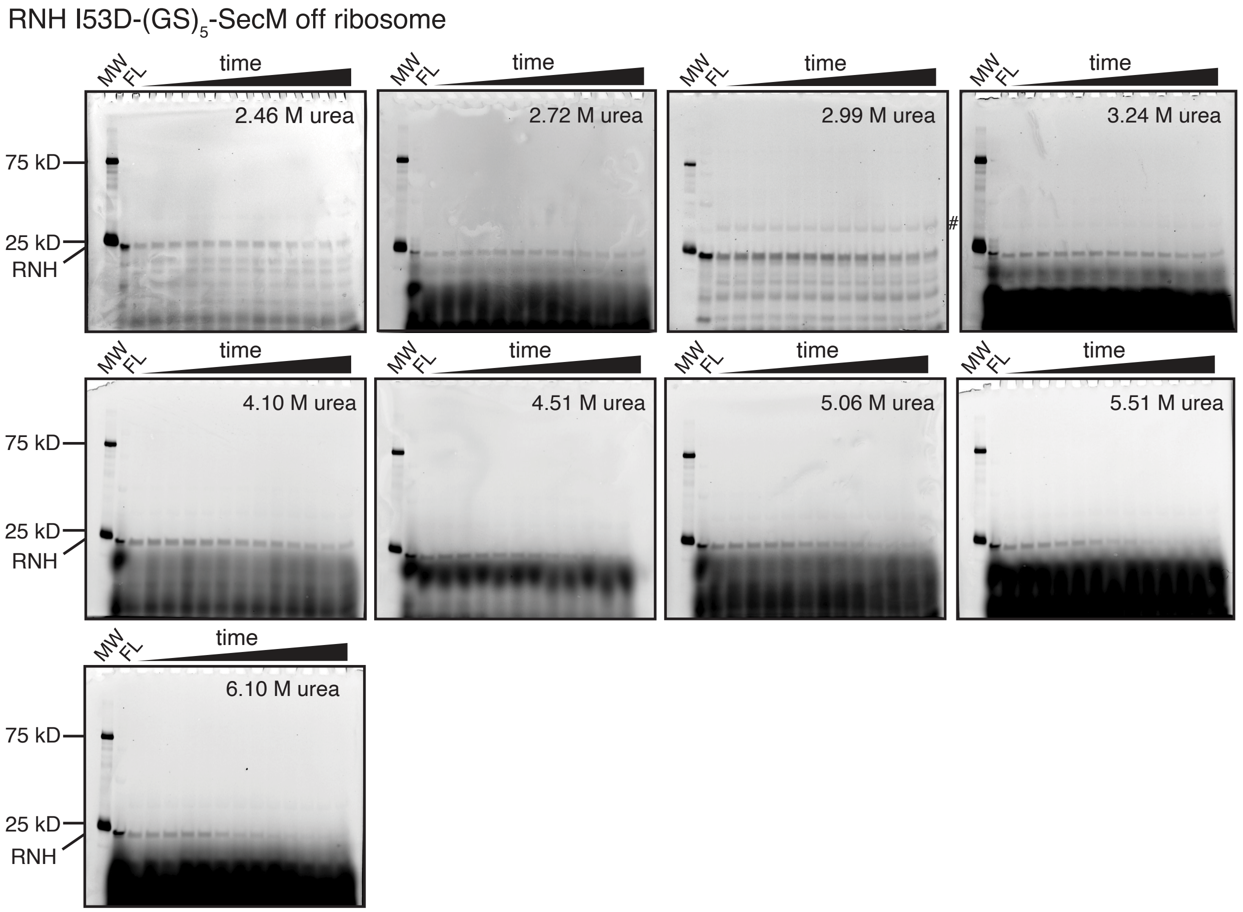

Figure S1 Gels for unfolding kinetics off the ribosome measured by pulse proteolysis. Pulse proteolysis of RNase H I53D-$(GS)_5$-SecM off the ribosome. # marks the electrophoretic band of RNase A. Time varies by sample. FL is a marker for the full-length RNase H.

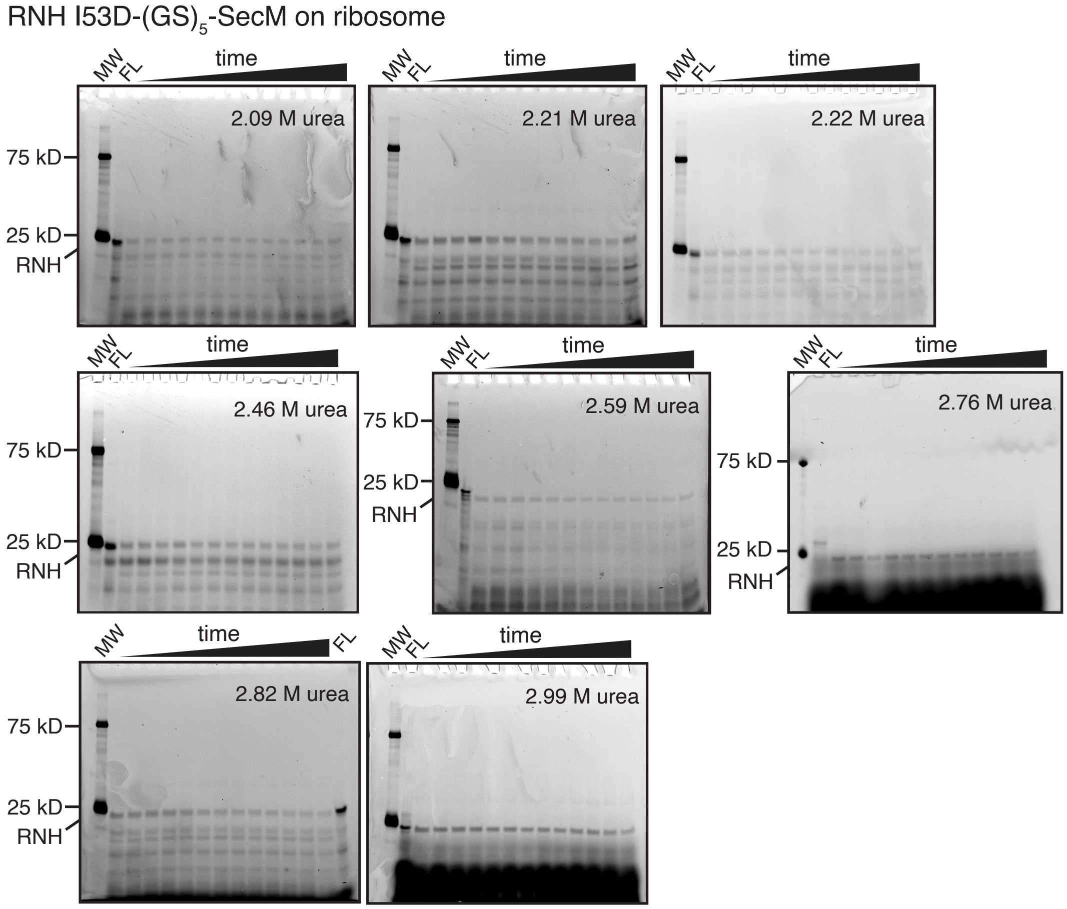

Figure S2 Gels for unfolding kinetics on the ribosome measured by pulse proteolysis. Pulse proteolysis of RNase H I53D-$(GS)_5$-SecM on the ribosome. Time varies by sample. FL is a marker for the full-length RNase H.
